# Linking mosquito surveillance to dengue fever through Bayesian mechanistic modeling

**DOI:** 10.1371/journal.pntd.0008868

**Published:** 2020-11-23

**Authors:** Clinton B. Leach, Jennifer A. Hoeting, Kim M. Pepin, Alvaro E. Eiras, Mevin B. Hooten, Colleen T. Webb

**Affiliations:** 1 Graduate Degree Program in Ecology, Colorado State University, Fort Collins, Colorado, United States of America; 2 Department of Statistics, Colorado State University, Fort Collins, Colorado, United States of America; 3 National Wildlife Research Center, United States Department of Agriculture, Wildlife Services, Fort Collins, Colorado, United States of America; 4 Departamento de Parasitologia, Universidade Federal de Minas Gerais, Belo Horizonte, Minas Gerais, Brazil; 5 U.S. Geological Survey, Colorado Cooperative Fish and Wildlife Research Unit, Fort Collins, Colorado, United States of America; 6 Department of Fish, Wildlife, and Conservation Biology, Colorado State University, Fort Collins, Colorado, United States of America; Institute for Disease Modeling, UNITED STATES

## Abstract

Our ability to effectively prevent the transmission of the dengue virus through targeted control of its vector, *Aedes aegypti*, depends critically on our understanding of the link between mosquito abundance and human disease risk. Mosquito and clinical surveillance data are widely collected, but linking them requires a modeling framework that accounts for the complex non-linear mechanisms involved in transmission. Most critical are the bottleneck in transmission imposed by mosquito lifespan relative to the virus’ extrinsic incubation period, and the dynamics of human immunity. We developed a differential equation model of dengue transmission and embedded it in a Bayesian hierarchical framework that allowed us to estimate latent time series of mosquito demographic rates from mosquito trap counts and dengue case reports from the city of Vitória, Brazil. We used the fitted model to explore how the timing of a pulse of adult mosquito control influences its effect on the human disease burden in the following year. We found that control was generally more effective when implemented in periods of relatively low mosquito mortality (when mosquito abundance was also generally low). In particular, control implemented in early September (week 34 of the year) produced the largest reduction in predicted human case reports over the following year. This highlights the potential long-term utility of broad, off-peak-season mosquito control in addition to existing, locally targeted within-season efforts. Further, uncertainty in the effectiveness of control interventions was driven largely by posterior variation in the average mosquito mortality rate (closely tied to total mosquito abundance) with lower mosquito mortality generating systems more vulnerable to control. Broadly, these correlations suggest that mosquito control is most effective in situations in which transmission is already limited by mosquito abundance.

## Author summary

The contribution of the mosquito vector *Aedes aegypti* to the spread of dengue fever depends not only on their abundance, but also on the likelihood of an exposed mosquito living long enough to incubate the dengue virus and subsequently transmit it to a susceptible human host. We developed a mechanistic model that accounts for the role of this process in the dynamics of dengue fever and fit the model to a time series of human case reports and mosquito trap counts from the city of Vitória, Brazil. We then used this fitted model to simulate the effect of mosquito control implemented at different times of the year and found that mosquito control leads to the largest reduction in human dengue cases over the following year when implemented in early September, during the dengue off-season. Further, the effectiveness of mosquito control was strongly negatively correlated with the overall average abundance of mosquitoes. Together with the timing of effective control, these results suggest that mosquito control is most effective when mosquitoes are already limiting to transmission.

## Introduction

Dengue fever is a massive global public health burden, with millions of cases per year [1]. Because the dengue virus (DENV) is transmitted by the mosquito *Aedes aegypti*, dengue fever is prevented primarily through mosquito control programs [2]. Though there have been documented successes, there is limited evidence for the long-term sustainability and effectiveness of these control programs [3]. As a result, there is a growing recognition that effective control needs to be guided by high quality vector surveillance, together with quantitative tools that synthesize vector surveillance with clinical surveillance, account for local epidemiology, and facilitate local decision making [3, 4]. Moreover, mosquito control needs to be guided by an understanding of the link between mosquito abundance and disease risk so that the mosquitoes most responsible for transmission can be targeted [4, 5].

Many of the attempts to establish this link have found a weak relationship between mosquito abundance indices and incidence of disease in humans [6–8]. However, these attempts often do not account for the complex, non-linear interactions that mediate the relationship between mosquito abundance and human disease. In particular, host immunity is a key intrinsic driver of infectious disease dynamics, and conditions favorable for transmission can only lead to an outbreak of disease when there is a sufficiently large population of susceptible hosts [9, 10]. As such, the ability of mosquitoes to contribute to DENV transmission depends critically on the level of immunity in the human population [5]. Further, the cycle of transmission between humans and mosquitoes is influenced not just by mosquito abundance, but also by mosquito survival relative to the virus incubation period in mosquitoes [11]. In fact, whether or not an exposed mosquito will survive long enough to become infectious represents a critical bottleneck in the transmission process and leads to nonlinear dependence of transmission on mosquito survival [11].

The importance of intrinsic nonlinearities, potentially alongside seasonality and stochastic forcing [9, 12, 13], in governing human disease risk highlights the need to integrate mechanistic modeling into the quantitative tools used to understand the effects of control interventions. Such mechanistic models can often perform better than complex autoregressive statistical models in describing and forecasting population dynamics [14]. Moreover, in the absence of case-control studies, mechanistic models can provide scenario-based tools that can be used to predict the effect of management actions [15].

Differential equation models provide a natural way to describe mechanistic processes, but that description must also account for sources of uncertainty [16, 17]. In particular, the values of parameters (e.g., the average length of time for which a host is infectious) are often uncertain, which can lead to large uncertainty about the effects of management actions [18]. The structure of the processes themselves can be uncertain [12], and needs to be informed by available, often noisy, data. Bayesian hierarchical modeling provides a coherent framework to account for and integrate this uncertainty across the three levels of the model (data, process, and parameters [19, 20]).

In what follows, we integrate these elements—a detailed mechanistic model of dengue transmission with a full Bayesian accounting of uncertainty—to better understand the interplay of forces governing dengue dynamics and their interaction with potential vector control interventions. We apply this framework to clinical and entomological surveillance data from the city of Vitória, Brazil. These data allow us to estimate a latent time series of mosquito mortality rates that modulate the transmission process and link mosquito abundance to human disease. We then use the fitted model to explore how perturbations to the mosquito population propagate and interact with the nonlinearities of dengue transmission to better inform mosquito control efforts.

## Methods

### Ethics statement

We did not obtain Institutional Review Board (IRB) approval for this work as the data we worked with were received by us as aggregated data at the weekly and neighborhood level. Hence, this research does not meet the definition of human subjects research requiring IRB approval. The data were analyzed in the aggregated form, which protects the anonymity of individuals.

### Study system and data

Vitória is a coastal city and the capital of the state of Espírito Santo, Brazil, with a population of 327,801 as of 2010 [21]. Since 2008, the company Ecovec has monitored mosquito abundance for the city using approximately 1327 sticky traps (MosquiTRAP, [22]) arranged in a roughly 250m grid across the city [7, 23]. Each trap is checked weekly and the mosquitoes inside counted and identified, with the results sent to a central database that city managers then use to map mosquito infestations and target control. These data comprise 243 weeks (week 1 of 2008 through week 34 of 2012) of total city-wide counts of trapped gravid female *Aedes aegypti*. It is important to note that this time series reflects both natural fluctuations in mosquito density and fluctuations driven by the city’s existing mosquito control program. In addition, dengue fever is a mandatory notifiable disease, and thus the city’s Ministry of Health Secretary maintains a database of weekly notified probable dengue cases (i.e., medical care sought for dengue-like symptoms) for the same time period.

### Process model

Dengue epidemiology is complicated considerably by the presence of four simultaneously circulating serotypes. Infection with one serotype confers life-long immunity to that serotype, along with temporary immunity to other serotypes [24]. As this cross-immunity wanes, antibodies from the previous infection can result in antibody-dependent enhancement (ADE), wherein human hosts are more susceptible to infection with the other serotypes and more likely to develop severe symptoms (i.e., dengue hemorrhagic fever or dengue shock syndrome) [24]. The strength and duration of these different inter-serotype interactions are not well understood, although different models suggest that temporary cross-immunity alone (without ADE) is sufficient to reproduce observed multi-annual dynamics in Thailand [24, 25].

Explicitly capturing the cross-immune interactions among all four serotypes, or even only two of the four [26], leads to a large and complex mechanistic model. Moreover, because dengue case reports do not identify serotype, there is not enough information in our data to inform the dynamics of individual serotypes. As such, we captured temporary cross-immunity, and the potential for multiple sequential infections, as simply and tractably as possible in a susceptible–exposed–infectious–recovered–susceptible (SEIRS) compartment model, similar to [27–30]. Although it captures the critical influence of temporary immunity, this framework does not account for the potential relationship between an individual’s infection history and the likelihood that a new infection will be symptomatic (and thus reported). In particular, secondary infections appear more likely to be symptomatic than primary infections [31, 32], while third and fourth infections appear much less likely to be symptomatic [33] (but see [34] who found similar rates of symptomatic cases across infection number). Despite these potential differences in reporting rate, modeling work has suggested that the dynamics of primary and secondary infections are closely coupled (and thus not dynamically distinct) under many conditions [35]. Moreover, given the short time scale of our data (5 years) relative to the period of cross-immunity (roughly 2 years [25]), we expect that third and fourth infections will be relatively rare. We thus expect the SEIRS framework, and the assumption of equal symptomatic rates across infection number, to be sufficient for capturing the dengue dynamics of Vitória and the relationship between mosquito abundance and human disease.

In the SEIRS framework, the total human population of Vitória (*N*) is divided into susceptible (*S*), exposed (*E*), infectious (*I*), and immune (*R*) classes. Susceptible humans (*S*) become exposed (*E*) through contact with infectious mosquitoes (*V*_*I*_). Following a latent period ($\frac{1}{\rho }$), exposed humans become infectious (*I*) at which point they can infect susceptible mosquitoes (*V*_*S*_). Infectious humans recover at rate *γ* and subsequently remain immune (*R*) for a period ($\frac{1}{\delta }$) after which they re-enter the susceptible class. Similarly, susceptible mosquitoes (*V*_*S*_) become exposed (*V*_*E*_) by biting infectious humans and pass through a temperature-dependent incubation period ($\frac{1}{\rho_{v}\left(t\right)}$) before becoming infectious (*V*_*I*_). Because the assumption of an exponentially distributed incubation period (implicit in the specification of a differential equation model) is a poor fit to laboratory observations [36], we instead implemented a gamma-distributed incubation period by chaining together multiple exposed classes (*V*_*Ej*_, taking advantage of the fact that a gamma-distributed random variable can be generated through the sum of exponential random variables with the same rate parameter) [37]. Total mosquito population size (*V*_*N*_) is controlled by a forced, seasonally varying growth rate (*r*(*t*)), while the transmission bottleneck is captured with a forced, seasonally varying mortality rate (*d*(*t*)). Captured mosquitoes (*V*_*C*_) accumulate at rate *ϕ*_*q*_*τ*(*t*), where *ϕ*_*q*_ is the per-trap capture rate, and *τ*(*t*) is the number of traps deployed in week *t*.

We specified the differential equations governing the human population as:

$$\frac{dS}{dt}=bN-bS-\lambda \frac{V_{I}}{N}S+\delta R$$ (1)

$$\frac{dE}{dt}=\lambda \frac{V_{I}}{N}S-\left(\rho +b\right)E$$ (2)

$$\frac{dI}{dt}=\rho E-(\gamma +b)I$$ (3)

$$\frac{dR}{dt}=\gamma I-(\delta +b)R$$ (4)

while the equations governing the mosquito (vector) population are:

$$\frac{dV_{N}}{dt}=r\left(t\right)V_{N}-\phi_{q}\tau \left(t\right)V_{N}$$ (5)

$$\frac{dV_{E1}}{dt}=\lambda \frac{I}{N}V_{S}-\left(4\rho_{v}\left(t\right)+d\left(t\right)+\phi_{q}\tau \left(t\right)\right)V_{E1}$$ (6)

$$\frac{dV_{E2}}{dt}=4\rho_{v}\left(t\right)V_{E1}-\left(4\rho_{v}\left(t\right)+d\left(t\right)+\phi_{q}\tau \left(t\right)\right)V_{E2}$$ (7)

$$\frac{dV_{E3}}{dt}=4\rho_{v}\left(t\right)V_{E2}-\left(4\rho_{v}\left(t\right)+d\left(t\right)+\phi_{q}\tau \left(t\right)\right)V_{E3}$$ (8)

$$\frac{dV_{E4}}{dt}=4\rho_{v}\left(t\right)V_{E3}-\left(4\rho_{v}\left(t\right)+d\left(t\right)+\phi_{q}\tau \left(t\right)\right)V_{E4}$$ (9)

$$\frac{dV_{I}}{dt}=4\rho_{v}\left(t\right)V_{E4}-\left(d\left(t\right)+\phi_{q}\tau \left(t\right)\right)V_{I}$$ (10)

$$\frac{dV_{C}}{dt}=\phi_{q}\tau \left(t\right)V_{N}$$ (11)

$$V_{S}=V_{N}-V_{E}-V_{I}.$$ (12)

We modeled the centered and log-transformed mosquito mortality rate (*ν*) and the per-capita mosquito growth rate (*r*) as forced harmonic oscillators with natural periods of one year:

$$\frac{d^{2}\nu }{dt^{2}}=-\omega^{2}\nu +\epsilon_{\nu t}$$ (13)

$$\frac{d^{2}r}{dt^{2}}=-\omega^{2}r+\epsilon_{rt},$$ (14)

where the angular frequency of the oscillator, *ω* = 2*π*/52, the mosquito death rate *d*(*t*) = *d*_0_ exp(*ν*(*t*)), and

$$\epsilon_{\nu t}∼\text{Normal}(0,\sigma_{\nu }^{2})$$ (15)

$$\epsilon_{rt}∼\text{Normal}(0,\sigma_{r}^{2}),$$ (16)

for each week *t* = 1, …, 243. These stochastically-forced harmonic oscillators provide a flexible framework for generating smooth seasonal oscillations in the latent mosquito processes [38].

### Data model

To connect the differential equation model to the observed case reports, we added an extra state, *C*, that collects the cumulative number of transitions from the exposed to infectious class (assuming that case reporting coincides with the onset of symptoms). We then modeled the number of new cases reported in week *t* (*y*_*t*_) as:

$$y_{t}∼\text{NegBin}\left(\phi_{y}\left(C\left(t\right)-C\left(t-1\right)\right),\eta_{y}\right),$$ (17)

where *ϕ*_*y*_ is the reporting probability, *C*(*t*) − *C*(*t* − 1) is the number of new infectious humans in week *t*, and *η*_*y*_ controls the overdispersion relative to the Poisson distribution.

We similarly modeled the number of mosquitoes trapped in week *t* (*q*_*t*_) as:

$$q_{t}∼\text{NegBin}\left(V_{C}\left(t\right)-V_{C}\left(t-1\right),\eta_{q}\right),$$ (18)

where *V*_*C*_(*t*) − *V*_*C*_(*t* − 1) is the number of new mosquitoes captured in week *t*, and *η*_*q*_ controls overdispersion relative to the Poisson distribution.

### Parameterization and priors

Several of the parameters in this model are assumed to be fixed and known (Table 1). The human population size and average life span (which we use to parameterize the birth/death rate) for Vitória were taken from the 2010 census. To maintain identifiability, the transmission rate (λ) was also fixed at literature values. Lastly, the extrinsic incubation period in mosquitoes was modeled as a function of weekly mean temperature and forced with weather station data obtained from WeatherUnderground [39].

**Table 1:** Model parameters and their values. The values in parentheses after the posterior means give the 80% credible interval. See S1 Text for a full description of all prior distributions.

Parameter	Description	Prior mean	Posterior mean	Citation
*N*	Human population size in Vitória, Brazil	327801		[21]
1/*d*	Human life-span	76 years		[40]
λ	Transmission rate	4.87 week^−1^		[41]
1/*ρ*_*v*_(*t*)	Extrinsic incubation period	$\frac{1}{7}\exp(7.9-0.21T(t))$ weeks		[36]
*V*_*E*0_	Initial exposed mosquitoes	0		
*V*_*I*0_	Initial infectious mosquitoes	0		
*d*_0_	Baseline mosquito mortality rate	1.47 week^−1^	0.88 (0.7, 1.1)	[42]
1/*ρ*	Latent period in host	0.87 weeks	1.72 (1.2, 2.3)	[36]
*γ*	Rate of loss of infectiousness	3.5 week^−1^	3.6 (3.2, 4.1)	[43]
1/*δ*	Period of cross-immunity	97 weeks	114 (72, 160)	[25]
*σ*_*r*_	Standard deviation of mosquito growth rate forcing	0*	0.013 (0.01, 0.02)	
*σ*_*ν*_	Standard deviation of mosquito mortality rate forcing	0*	0.0005 (0.0004, 0.0008)	
*S*_0_	Proportion initially susceptible	0.4	0.42 (0.28, 0.57)	[44]
*E*_0_	Number initially exposed	100	148 (104, 196)	
*I*_0_	Number initially infectious	60	79 (45, 116)	
*r*_0_	Initial mosquito population growth rate	0	0.008 (-0.01, 0.02)	
*ν*_0_	Initial unconstrained mosquito mortality rate	0	-0.16 (-0.4, 0.08)	
*V*_*N*0_	Initial mosquito population size	2*N*	1.5*N* (1.1*N*, 1.9*N*)	
*ϕ*_*y*_	Reporting probability	0.083	0.14 (0.1, 0.18)	[45]
log(*ϕ*_*q*_)	Log per-trap mosquito capture rate	−13	-13.2 (-13.6, -13)	
*η*_*y*_	Overdispersion of case reports	0*	0.12 (0.1, 0.13)	
*η*_*q*_	Overdispersion of mosquito trap counts	0*	0.14 (0.12, 0.16)	

* indicates prior mode, rather than mean.

The remaining parameters include the epidemiological parameters controlling the average latent, infectious, and immune periods (*ρ*, *γ*, *δ*) and average mosquito lifespan (*d*_0_), the initial conditions of the model (*S*_0_, *E*_0_, *I*_0_, *R*_0_, *V*_*N*0_, *ν*_0_, *r*_0_), the variances of the latent mosquito processes ($\sigma_{r}^{2}$, $\sigma_{\nu }^{2}$), and the remaining measurement parameters (*ϕ*_*y*_, *ϕ*_*q*_, *η*_*y*_, *η*_*q*_). Where possible, we specified informative prior distributions for these parameters based on existing laboratory and field studies (see Table 1 for means and S1 Text for detailed explanations).

### Implementation

Combining the data, process, and parameter models [19], we summarize the full hierarchical model as:

$$y_{t}|\cdot ∼\text{NegBin}(\phi_{y}\left(C\left(t\right)-C\left(t-1\right)\right),\eta_{y}),$$ (19)

$$q_{t}|\cdot ∼\text{NegBin}(V_{C}\left(t\right)-V_{C}\left(t-1\right),\eta_{q})$$ (20)

$$\left(C,V_{C}\right)=M(\epsilon_{r},\epsilon_{\nu },\theta )$$ (21)

$$\epsilon_{rt}∼\text{Normal}(0,\sigma_{r}^{2})$$ (22)

$$\epsilon_{\nu t}∼\text{Normal}(0,\sigma_{\nu }^{2})$$ (23)

θ∼[θ] (24)

where ***θ*** is a vector of all the model parameters and initial conditions, and $M(\epsilon_{r},\epsilon_{\nu },\theta )$ represents the (numeric) solution to the differential equation model (Eqs 1–14) as a function of ***θ*** and the weekly stochastic forcing terms (***ϵ***_***r***_, ***ϵ***_***ν***_). Sampling from the posterior distribution of the parameters in a mechanistic model is difficult due to multimodality, variable parameter sensitivities (e.g., small changes in one parameter may lead to large changes in output, while similar changes in another parameter may have little effect), and potentially strong posterior correlations induced by the nonlinearity of the differential equation model [14, 46, 47]. However, the variable *ϵ*_*t*_ introduces flexibility to the mechanistic model that remedies lack-of-fit when the process parameters are far from optimal [48], thereby reducing multimodality and helping to smooth the posterior surface. Gradient-based methods like Hamiltonian Monte Carlo (HMC) can then more easily and efficiently traverse the posterior. Samples from the posterior distribution were generated using HMC implemented in the rstan package [49, 50] for R [51]. We ran 3 chains with different starting values for 4,000 iterations each, discarding the first 2,000 as burn-in. Convergence diagnostics and mixing were evaluated using the shinystan package [52]. In our implementation, the solution to the differential equation model was approximated with an Euler scheme with a time step of 1 day. Timesteps as small as 1/8 of a day were explored and did not qualitatively change the modeled dynamics. Code is available from https://github.com/clint-leach/mosquito-recon.

### Mosquito control simulations

Given a subset of the samples from the posterior distribution as obtained above (2000, taken to reduce computation time), we simulated the effects of a single pulse of mosquito control applied in each week of the first three years of the time series. Because the city already implements responsive, targeted control with the aim of reducing local mosquito density during an existing outbreak, we focused our simulations on exploring the longer-term feedbacks induced by mosquito control and the ability of an intervention to reduce the disease burden over the following year. For each week and each posterior sample, we simulated the dynamics resulting from a 5% reduction in the mosquito population implemented at the beginning of that week (affecting susceptible, exposed, and infectious mosquitoes equally). To capture the likely rapid rebound in mosquito abundance following a single pulse of control [28, 53], we also simulated a 5% increase in mosquito birth rate in the following week to return mosquito abundance to its previous trajectory (without this, the 5% reduction in abundance persists indefinitely). We then compared the number of cases produced over the year following the control intervention in the control scenario to the same number in the uncontrolled scenario. A 5% reduction in mosquito abundance was chosen to keep our simulations conservative relative to field estimates of the mortality induced by spraying [54], and to avoid pushing the model into the unrealistic range of dengue eradication.

## Results

**Fig 1:**
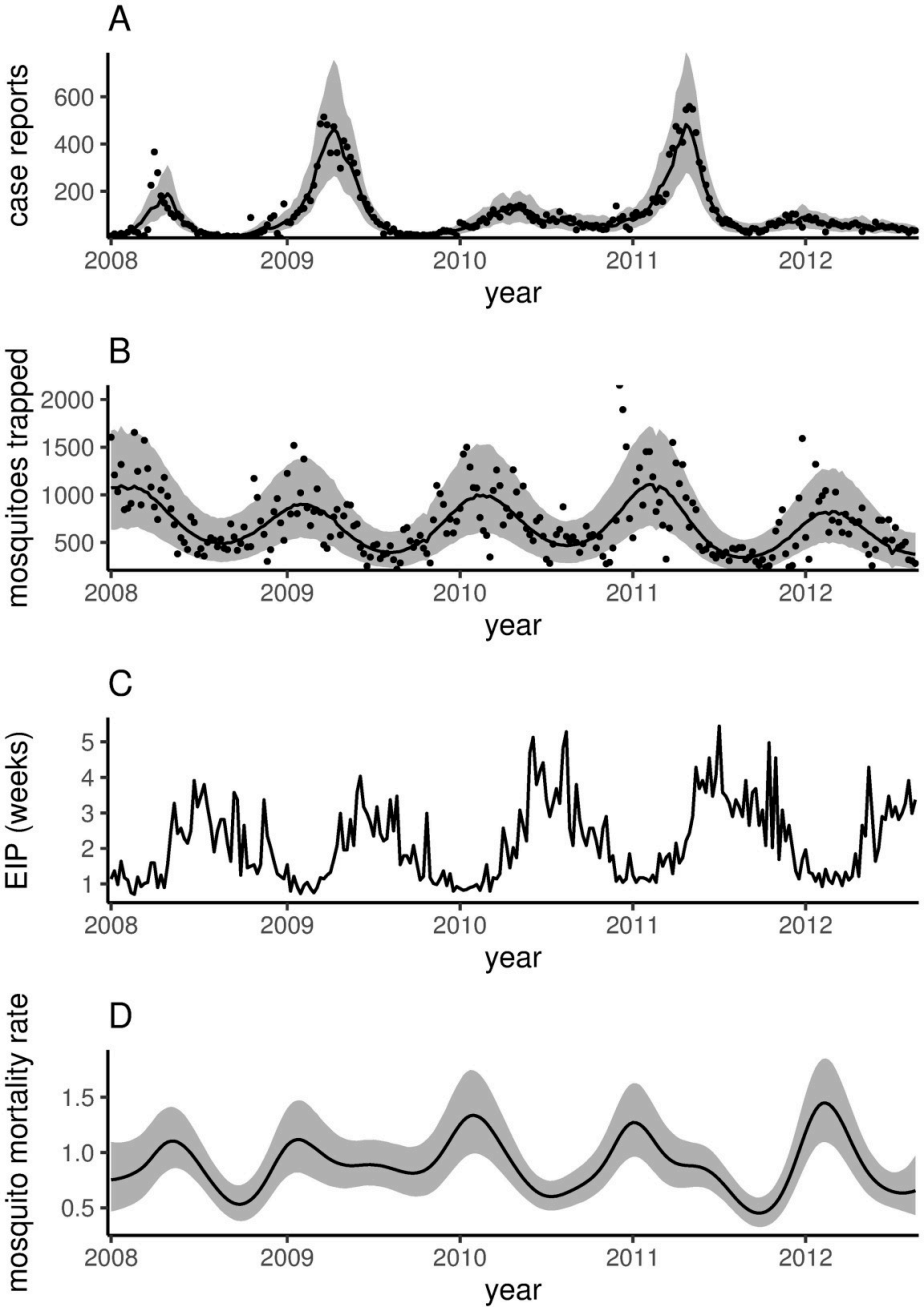
Vitória data and model estimates. A: weekly observed case reports (points), with corresponding posterior median (black line) and 80% posterior credible interval (gray band). B: weekly mosquito trap counts (points), with posterior median (black line) and 80% posterior credible interval (gray band). C: extrinsic incubation period (EIP; weeks), computed from weekly mean temperature data. D: estimated weekly mosquito mortality rate, with the posterior median (black line) and the 80% posterior credible interval (gray band).

The model captured the observed dynamics of both case reports and mosquito trap counts (Fig 1). The estimated posterior median case reports explained 91% of the variation in the observed time series, while the posterior median mosquito trap counts explained 46% of the variation in the observed time series. In addition, posterior predictive checks showed that the model reproduced the total number of cases reported and mosquitoes captured as well as the autocorrelation structure of both time series (with the exception of slightly underestimating the autocorrelation for short lags, S6, S7 and S8 Figs). The posterior distributions of the rate of infectious decay (*γ*) and the period of cross-immunity (1/*δ*) did not differ substantially from their priors, suggesting that the Vitória data contained little additional information about these parameters (S3 Fig). The estimated latent period in a human host (1/*ρ*, the expected time it takes for an exposed human to become infectious to biting mosquitoes) was influenced more strongly by the data, with a posterior mean of 1.73 weeks compared to a prior mean of 0.87 weeks. Further, the posterior mean case reporting rate (*ϕ*) was 0.14, larger than the prior mean of 0.08.

**Fig 2:**
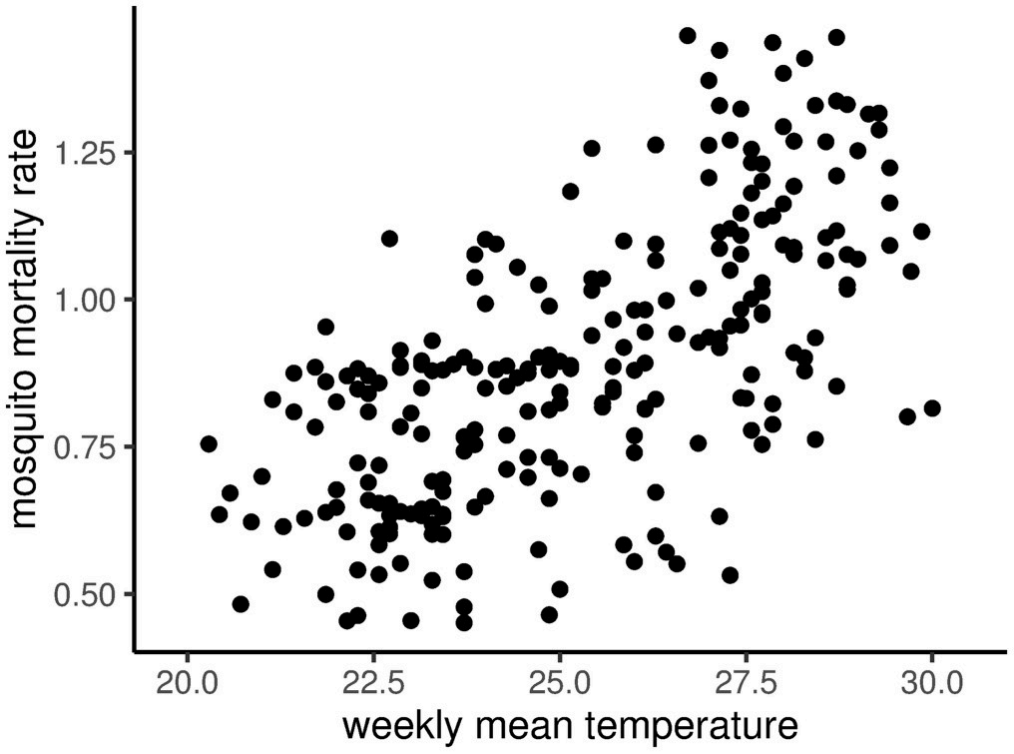
Mosquito mortality and temperature. Posterior median mosquito mortality rate as a function of weekly mean temperature (degrees Celsius).

The estimated weekly mosquito mortality rate varied seasonally, with generally high mortality early in the year and low mortality in August to October (Fig 1). This seasonal trend broadly tracked seasonal variation in temperature (Fig 2, correlation coefficient of 0.64) and mosquito trap counts (Fig 1), though the shape of the annual trajectory differed from year to year. The posterior distribution of the baseline mortality rate (*d*_0_) had a mean of 0.88/week, roughly 60% of the prior mean. The marginal posterior means of the *ϵ*_*νt*_ forcing the mosquito mortality process exhibited a higher-frequency periodic oscillation (S1 Fig), although the marginal posterior distribution of each *ϵ*_*νt*_ overlapped zero. In addition, the standard deviation of the mortality forcing terms was small relative to the weak prior (*E*(*σ*_*ν*_|**y**) = 0.01, S2 Fig).

**Fig 3:**
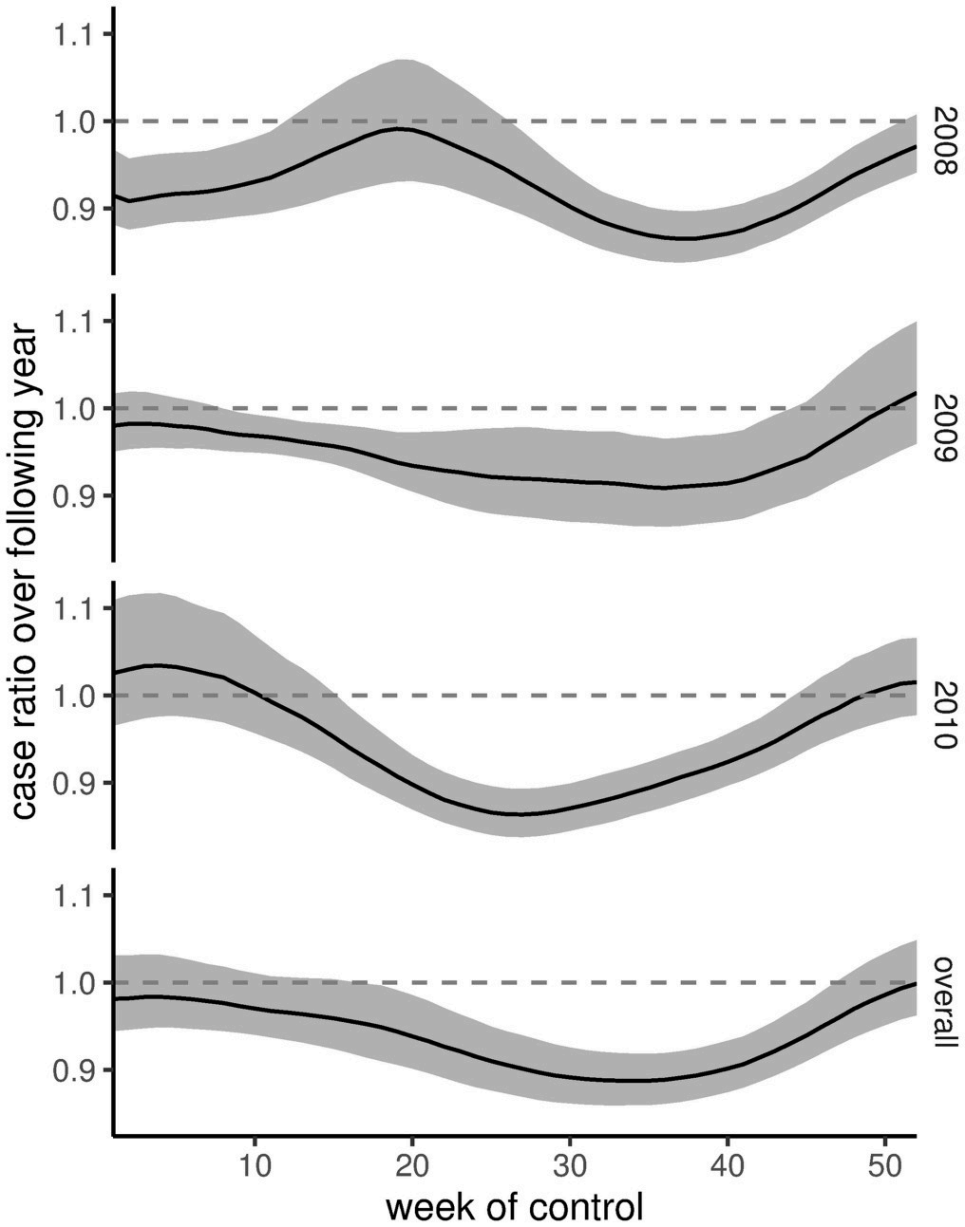
The effect of mosquito control as a function of the week in which it was applied. Summary of the posterior predicted effect of mosquito control implemented in a given week of the year on the number of cases in the following year (relative to the number of cases expected without control). Black lines indicate the posterior median, while gray ribbons indicate the 80% credible interval. The first three panels show the results for control implemented in the years 2008-2010, and the last panel shows the overall effect of control impelmented in a given week of the year, summing over all three years. For example, mosquito control applied in week 37 of 2008 would have prevented about 13% of the human cases over the following year (i.e., the caseload would have been 87% of the expectation without control).

The effect of a given mosquito control intervention (i.e., the temporary removal of 5% of the adult population in a given week) on the number of cases in the following year (relative to no control) varied both seasonally and interannually (Fig 3). This variation in the effect of control was tightly correlated with the estimated mosquito mortality, with a median posterior correlation between the two time series of 0.96. As such, the seasonal variation in the effectiveness of control followed the same trend as mosquito mortality rate (although the annual minima in the case ratio time series generally fell 1 to 2 weeks before the minima in the mosquito mortality time series), with the largest reductions in case load resulting from interventions during the dengue off-season (early September for 2008 and 2009, and mid-July for 2010). Summing over the interannual variation to compute the overall effect of control implemented in a given week of the year, we found that mosquito control was most effective when implemented around week 34 (late August/early September), reducing the estimated case load by roughly 14% (Fig 3). This broadly corresponds to the end of the dry season in Vitória, when both dengue case reports and mosquito trap counts are low.

**Fig 4:**
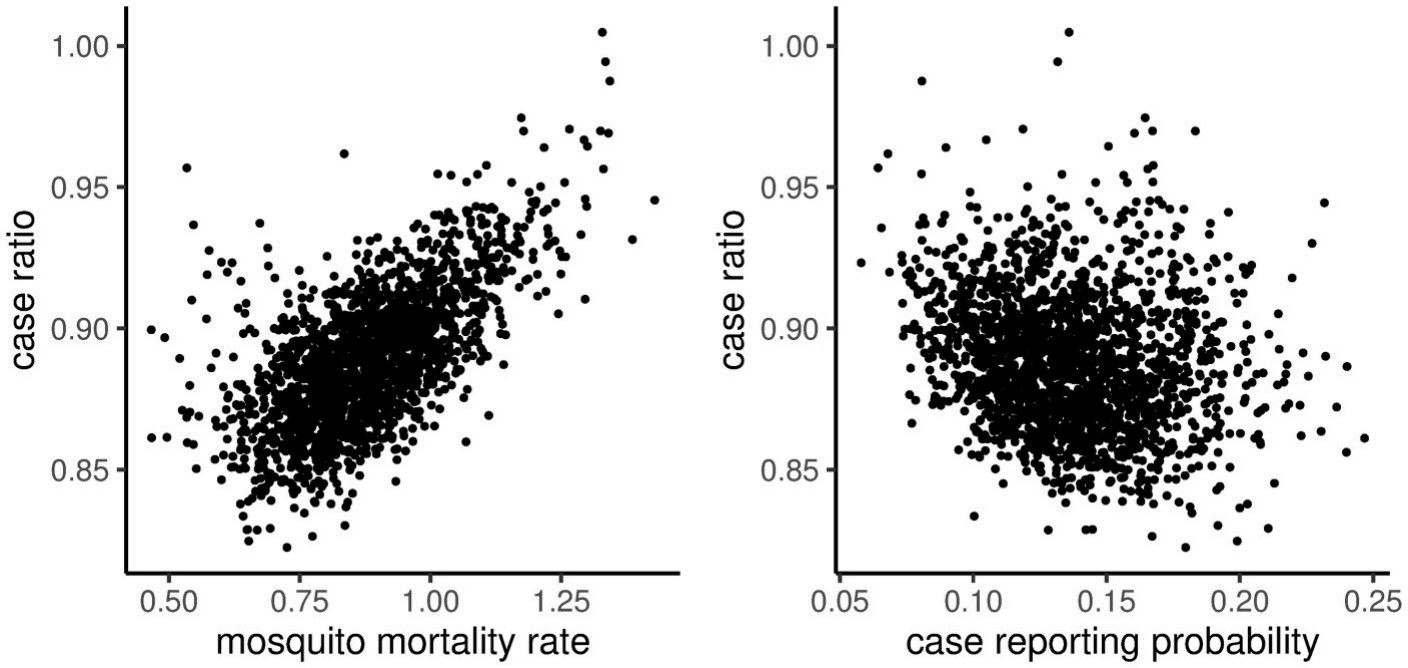
Posterior correlation between system parameters and the effectiveness of control. The y-axis represents the effectiveness of optimally timed control, i.e., the effect of control implmented in the 34th week of the year on the relative number of cases in the following year, summed over 2008, 2009, and 2010. Each point represets a single sample from the posterior distribution, giving the number of cases in the controlled simulation (relative to the number of cases expected without control) as a function of A: the mean mosquito mortality rate, *d*_0_, and B: the case reporting probability, *ϕ*, from that posterior sample.

The variation associated with the posterior predicted effect of control implemented in week 34 of the year (i.e., the width of ribbon in Fig 3) was correlated with the baseline mosquito mortality rate (*d*_0_, posterior correlation coefficient of 0.65) and the case reporting probability (*ϕ*, posterior correlation coefficient of -0.19). Simulated mosquito control created the largest reduction in case reports in posterior samples with low baseline mosquito mortality rate and/or high reporting probability, while control was relatively less effective in simulations from samples with high mosquito mortality or low reporting probability (Fig 4). Thus mosquito control was more effective at reducing disease burden in simulations with long average mosquito lifespans (i.e., low mosquito mortality rates) or low overall prevalence (i.e., fewer undetected cases).

## Discussion

### Processes driving effect of mosquito control

The dynamics of dengue fever, like those of many infectious diseases [9, 12] and ecological systems [55], are driven by the combined efforts of intrinsic non-linearities, seasonality, and stochasticity. Seasonality, in particular, is an important factor in capturing the annual cycle of dengue outbreaks [24, 25, 56]. However, the observed seasonality in transmission likely emerges from the combined effects of multiple seasonally-varying components that may be driven by different environmental factors that oscillate in different phases (e.g., mosquito abundance seems to lag slightly behind temperature-driven variation in extrinsic incubation period, Fig 1). Integrating these seasonally-varying components into a synthetic measure of transmission potential (e.g., a temperature-dependent effective reproduction number, [57]), or more specifically, a measure of the transmission potential of mosquitoes, is difficult.

We positioned the latent mosquito mortality as the link between mosquito abundance, the extrinsic incubation period, and human cases. In this way, the estimated mosquito mortality rate serves as an index of transmission potential, opening or closing the mosquito life history bottleneck [11] as necessary to fit to the case reports data. The resulting seasonality in the estimated trajectory suggests that the seasonality in mosquito abundance and the extrinsic incubation period was not sufficient to capture the observed case reports. In particular, when mosquitoes were relatively scarce and transmission limited, we estimated a relatively low mosquito mortality, suggesting that long-lived mosquitoes were required to maintain observed levels of transmission through the off-season. On the other hand, when mosquitoes were abundant, we estimated relatively high mosquito mortality rates, suggesting that transmission needed to be damped.

The importance of mosquito longevity in driving disease dynamics highlights the potential effectiveness of control efforts that target adult mosquitoes and disrupt transmission by preventing mosquitoes from living long enough to progress through the extrinsic incubation period to the infectious state and bite a susceptible human. In fact, this forms the basis for much of the theory of adult mosquito control [3, 11, 28]. The high correlation between our estimated mosquito mortality and the effect of control confirms this theory, suggesting that control is most effective when it targets long-lived mosquitoes. Specifically, our simulated mosquito control interventions were most effective at reducing the disease burden when applied around week 34 (i.e., early September), in the dengue off-season. The effectiveness of this control was likely driven by the fact that transmission during the off-season was already limited by low mosquito abundance and a relatively high extrinsic incubation period. Given that limitation, transmission was maintained by relatively few long-lived mosquitoes, making the system vulnerable to perturbation.

On the other hand, we found that a single pulse of control was relatively less effective when implemented during an outbreak, when mosquitoes were abundant (and unlikely to be limiting transmission) but short-lived. Given the relatively high mosquito mortality rates during this time, exposed and infectious mosquitoes were already fairly ephemeral, such that the relatively small disruption induced by control likely made little difference. Moreover, due to the large number of infectious human hosts available to transmit to the remaining (and rapidly rebounding) mosquito population, the population of exposed mosquitoes likely recovered quickly [27, 28]. Barsante *et al.* [58] and Oki *et al.* [59] similarly found that control was most effective when applied well before peak prevalence, either during the dry season [58] or early in the rainy season [59] (September is near the end of the dry season in Vitória). While the immediate effects of mosquito control implemented during the decline phase of an outbreak may be masked by the natually fading transmission intensity [60], our results nonetheless indicated that disrupting inter-seasonal transmission can be an effective longer-term strategy [61]. Further, although large pulses of imported cases could potentially swamp the effects of early control, in additional simulations we found that our results were robust to the import of 10 infectious humans (roughly the same order as the number of locally reported cases) just before the annual outbreak.

The Bayesian framework allowed us to account for uncertainty across the data, process, and parameter levels of our model [19]. We carried this uncertainty through to our simulations of control interventions [18] and found that there was substantial uncertainty in the proportion of cases prevented by a control intervention (i.e., the width of the ribbons in Fig 3). Much of this uncertainty could be attributed to posterior uncertainty in the case reporting rate (*ϕ*) and the average mosquito mortality rate (*d*_0_). Specifically, we found that control implemented at the overall optimum (week 34) had the largest impact (i.e., the lowest case ratio) in simulations with a low average mosquito mortality and/or a high case reporting rate (Fig 4). Mosquito mortality and the case reporting rate were correlated with the overall level of mosquito abundance and the overall size of the susceptible population, respectively, suggesting that control was most effective in simulations with fewer mosquitoes and more susceptible humans. Hladish *et al.* [61] similarly found that simulated indoor residual spraying was more effective when the modeled mosquito abundance was already low. This suggests that control efforts that reduce the ability of mosquitoes to transmit DENV are most effective for situations in which mosquito abundance is already the limiting component to maintaining transmission (relative to other factors like human immunity).

The posterior correlation between the effectiveness of control, the case reporting rate, and the average mosquito mortality rate emphasizes that the impact of mosquito control is jointly regulated by both mosquito population dynamics and human immune processes. Similar observations were made by ten Bosch *et al.* [62] who found that models with longer periods of cross-immunity (such that susceptibles replenished more slowly) generated systems in which transmission was more difficult to disrupt with control actions. As a result of these relationships, efficient deployment of mosquito control, and accurate prediction of its effects, is likely to depend in part on our ability to monitor and predict the dynamics of human immunity. To meet these needs, existing mosquito monitoring efforts need to be paired with more detailed clinical surveillance [3] and tighter estimates of the period of cross-immunity [62] and the number of unreported cases [45]. In the absence of, or as a supplement to, such data, mechanistic models like the one developed here, or so-called TSIR (Time-series Susceptiple-Infected-Recovered) frameworks that reconstruct the dynamics of the susceptible class [25, 63], need to be further developed to better inform and understand mosquito control efforts.

### Interpretation of the estimated mosquito mortality rate

The positive correlation between the estimated mosquito mortality rate and the simulated effect of control suggests that mosquito control was most effective when it targeted long-lived mosquitoes during the inter-epidemic periods when mosquito abundance was low. However, the fact that the mosquito mortality forcing terms (*ϵ*_*ν*_, Eqs 13 and 16) were the only source of variability in the transmission process implies that the estimated mosquito mortality time series could have absorbed other sources of stochasticity or model misspecification. Hooker and Ellner [64] provide a framework for diagnosing such model misspecification in differential equation models using forcing functions similar to our implementation of the *ϵ*_*ν*_. In that framework, Hooker and Ellner [64] estimate nonparametric forcing functions that modify a fitted differential equation model to provide a good fit to the data. These forcing functions serve as residuals on the time derivatives, and can be more readily interpreted as indicators of lack-of-fit than residuals on the state variables [16, 64]. We do not employ the same explicit goodness-of-fit testing framework as [64], but we can inspect our estimated *ϵ*_*ν*_ forcing terms in the same spirit.

The periodic structure in the time series of the posterior means of the *ϵ*_*ν*_ (S1 Fig) suggests that these terms were accounting for more than just noise, and there may have been some unmodeled process influencing fluctuations in mosquito mortality and/or transmission. Following Hooker and Ellner, we can explore whether this process is likely to result from misspecification of the rates of change of the existing state variables (indicated by a dependence of *ϵ*_*νt*_ on other state variables), or from missing state variables altogether (indicated by an additional dependence of *ϵ*_*νt*_ on its own lagged values). The lack of any apparent relationship between the forcing terms and any of the estimated state variables, combined with the dependence of *ϵ*_*νt*_ on previous values (as apparent through the periodic structure), suggest that unmodeled state variables may be the more likely driver of model misspecification. These unmodeled components could include additional mosquito population dynamic processes (e.g., aquatic stage dynamics, environmental drivers, or control interventions), or epidemiological processes (e.g., multiple circulating serotypes of the dengue virus, subsets of the population with different mixing or risk levels).

Despite these potential sources of model misspecification, our estimated mosquito mortalities nonetheless fell within the reasonable range from the literature [42, 65]. Moreover, the fact that our estimated mortality rate increased with temperature also broadly agrees with the empirical literature on mosquito survival [42, 66, 67]. This suggests that regardless of unexplained structure in the forcing terms, the pattern of case reports was still very well described by realistic seasonal fluctuations in the mosquito mortality rate. As demonstrated by Reiner *et al.* [68] for malaria transmission, estimates of transmission potential can be sensitive to fluctuations in mosquito abundance and age structure. Moreover, given the broad importance of seasonality in understanding dengue epidemiology [62], and the role of mosquito mortality and age in driving the effect of control interventions, future work should focus on developing a more complete, predictive understanding of the seasonal drivers of mosquito mortality (including, potentially, control itself).

### Additional considerations and extensions

In addition to epidemiological complexity, dengue dynamics are further complicated by the reporting process. We estimated a relatively long average intrinsic incubation period (1/*ρ*, posterior mean of 1.7 weeks) relative to our prior mean (0.87 weeks), suggesting possible reporting delays [69]. Moreover, the case data to which we fit the model represent reports of “dengue-like illness,” without laboratory confirmation, and as such could include cases of other diseases with similar symptoms (e.g., chikungunya or Zika). However, neither chikungunya nor Zika had emerged as substantial public health threats in Brazil by the end of our time series in late 2012 [70, 71]. In addition, given the high underreporting rate expected for dengue fever [45], and the uncertainty incorporated into the measurement model, we expect misreported cases to have a small effect on our analyses.

Mosquito control interventions can prevent cases by acting on any of the components of vectorial capacity. We focused on the direct effect of killing adult mosquitoes on transmission, but adult control can also act by reducing egg laying and the number of mosquitoes in the next generation [72]. Given the relative simplicity of our mosquito model, we were unable to explore the feedbacks that adult control may induce in mosquito population dynamics, and instead assumed that mosquito populations quickly rebound from any perturbations [53]. We expect that capturing these feedbacks would likely reinforce our conclusions about the utility of off-season control, as the disruption to mosquito population dynamics would more strongly limit the ability of the mosquito population to maintain transmission through the off-season.

Although our results suggest that a pulse of adult control in the off-season may be an effective tool for preventing human cases, achieving particular control thresholds or policy goals will likely require deploying a combination of control interventions [72]. In fact, it is important to note that the data to which we fit our model implicitly reflect the control efforts already enacted by the city (the effects of which may have influenced our estimates of mosquito demographic rates). Thus, our mosquito control simulations should be interepreted as exploring the effect of an additional pulse of city-wide control in addition to the existing control activities. These control efforts are guided by the MI-Dengue system, which uses the trap-level mosquito surveillance data to target areas of high mosquito infestation for control (including source reduction, larvacide, and adulticide) [22]. Although these targeted, reactive interventions are necessary to help reduce local disease risk at times and locations of high mosquito abundance [73], our results suggest that an additional pulse of proactive control in the off-season when mosquitoes are less abundant would minimize human cases.

The frequent use of spatially-targeted mosquito control highlights the potential for spatial heterogeneities in disease risk within a city. In particular, structured human movement within the city is likely to induce heterogeneous human-mosquito mixing [74–76]. In addition, spatial variability in socioeconomic factors within the city may also modulate the extent to which mosquitoes in different parts of the city contribute to disease spread [77–80]. Although our model does not account for these heterogeneities, it was nonetheless able to capture the city-wide dynamics well, suggesting that there may be sufficient mixing to appear homogeneous at the city scale. Further, although our mechanistic model may be able to suggest when a city-wide intervention is likely to be effective, to make the best use of limited resources, spatial prioritization may still be necessary.

### Conclusions

Efforts to connect mosquito abundance to human disease are often hampered by the confounding influences of human immunity and mosquito survival. The challenges presented by these confounding factors highlight the value of mechanistic information in studying the effect of mosquito control on disease spread. In the Bayesian context we deployed, this mechanistic knowledge, as formalized in the specification of a differential equation model, can be viewed as part of the prior knowledge on the relationship between mosquito abundance and human disease [12, 17]. As such, we should neither ignore this mechanistic information, nor encode it so rigidly that it overwhelms the signal in our data.

We developed a simple yet realistic mechanistic model of dengue fever spread that represents the fundamental elements of our prior understanding of dengue epidemiology, while also allowing for uncertainty and flexibility in the fluctuations of mosquito demographic rates. This mechanistic framework allowed us to capture the critical contribution of long-lived, off-season mosquitoes to the maintenance of transmission and to identify critical intervention points that would not be apparent otherwise. The fully hierarchical Bayesian framework in which we embedded the mechanistic model allowed for a thorough accounting of uncertainty that was carried through to the evaluation of different control strategies. This combination of model features helps to meet the need for more effective, biologically grounded, and data-driven dengue control policies and offers a building block on which these tools can be further developed in the future.

## Supporting information

S1 Fig Posterior estimates of harmonic oscillator forcing functions. A: mosquito mortality forcing. B: mosquito growth forcing. Median posterior estimate (black line) and 80% credible interval (gray band).
(EPS)

S2 Fig Posterior distribution of variances on forcing terms. A: mosquito mortality forcing. B: mosquito growth forcing.
(EPS)

S3 Fig Posterior distribution of epidemiological parameters. The black line indicates the prior density. A: scaled latent period in human host (1/*ρ*). B: scaled rate of infectious decay in human host (*γ*). C: scaled period of cross-immunity (1/*δ*). D: scaled baseline mosquito mortality rate (*d*_0_).
(EPS)

S4 Fig Posterior distribution of initial conditions. The black line indicates the prior density. A: initial proportion of the human population that is susceptible. B: initial number of exposed individuals. C: initial number of infectious individuals. D: log initial number of mosquitoes-per-person. E: initial value of centered mosquito mortality rate oscillator. F: initial value of mosquito population growth rate oscillator.
(EPS)

S5 Fig Posterior distribution of measurement parameters. The black line shows the prior density. A: log of the mosquito trap capture rate (which is strongly correlated with initial mosquito abundance). B: human case reporting probability. C: mosquito trap count measurement over-dispersion (prior density too low to be visible). D: case report measurement over-dispersion (prior density too low to be visible).
(EPS)

S6 Fig Posterior check of autocorrelation structure in data. A: autocorrelation in case report time series. B: autocorrelation in trapped mosquito time series. Points indicate the observed autocorrelation at the given lag, and vertical lines give the 80% posterior credible interval of the autocorrelation in the estimated time series.
(EPS)

S7 Fig Posterior distribution of the total number of cases reported (A) and the total number of mosquitoes captured (B). The thick line indicates the observed totals.
(EPS)

S8 Fig Posterior distributions of the minimum and maximum weekly reported cases (A and C) and trapped mosquitoes (B and D). The thick line indicates the observed minima and maxima.
(EPS)

S9 Fig Posterior estimates of the mosquito mortality rate and the probability of a mosquito surving the external incubation period. The black line indicates the posterior median, while the gray ribbon shows the 80% credible interval. The probability of surviving the extrinsic incubation period (bottom panel) was computed by taking the ratio of the number of mosquitoes that entered the infectious class in a week over the total number of mosquitoes that exited the exposed class that week. That is, the probability of surviving the extrinsic incubatio period represents the fraction of mosquitoes leaving the exposed class that enter the infectious class.
(EPS)

S1 Text Full specification of the model and prior distributions. (PDF)
