# Supplementary material for: Linking mosquito surveillance to dengue fever through Bayesian mechanistic modeling
Source: PLoS Negl Trop Dis. 2020 Nov 23;14(11):e0008868. doi: 10.1371/journal.pntd.0008868 (PMC7721181; doi:10.1371/journal.pntd.0008868)
Supplement: S1 Text — (PDF) [file pntd.0008868.s010.pdf]

Supplementary text

Clinton Leach

November 4, 2020

Bayesian mechanistic model for dengue and mosquito dynamics

Note that in practice, we rescale the model described in the main text by human population size to keep state variables on a unit scale. This also rescales mosquito population size to number of mosquitoes per person. Adopting this rescaling, we can express the model as:

$$
\begin{aligned}
y_t &\sim \text{NegBin}(\phi_y(C_t - C_{t-1})N, \eta_y) && (1)\\
q_t &\sim \text{NegBin}((V_{Ct} - V_{Ct-1})N, \eta_q) && (2)\\
& && (3)\\
\frac{dC}{dt} &= \rho E && (4)\\
\frac{dS}{dt} &= b - bS - \lambda V_I S + \delta R && (5)\\
\frac{dE}{dt} &= \lambda V_I S - (\rho + b)E && (6)\\
\frac{dI}{dt} &= \rho E - (\gamma + b)I && (7)\\
\frac{dR}{dt} &= \gamma I - (\delta + b)R && (8)\\
& && (9)\\
\frac{dV_N}{dt} &= rV_N - \phi_q \tau(t) V_N && (10)\\
\frac{dV_{E1}}{dt} &= \lambda I V_S - (4\rho_v(t) + d(t) + \phi_q\tau(t))V_{E1} && (11)\\
\frac{dV_{E2}}{dt} &= 4\rho_v(t)V_{E1} - (4\rho_v(t) + d(t) + \phi_q\tau(t))V_{E2} && (12)\\
\frac{dV_{E3}}{dt} &= 4\rho_v(t)V_{E2} - (4\rho_v(t) + d(t) + \phi_q\tau(t))V_{E3} && (13)\\
\frac{dV_{E4}}{dt} &= 4\rho_v(t)V_{E3} - (4\rho_v(t) + d(t) + \phi_q\tau(t))V_{E4} && (14)\\
\frac{dV_I}{dt} &= 4\rho_v(t)V_{E4} - (d(t) + \phi_q\tau(t))V_I && (15)\\
\frac{dV_C}{dt} &= \phi_q\tau(t)V_N && (16)\\
V_S &= V_N - V_E - V_I && (17)\\
& && (18)\\
d(t) &= d_0 \exp(\nu(t)) && (19)\\
\frac{d^2\nu}{dt^2} &= -\omega^2\nu + \epsilon_{\nu t} && (20)\\
\frac{d^2 r}{dt^2} &= -\omega^2 r + \epsilon_{rt}, && (21)
\end{aligned}
$$

where $\omega = 2\pi/52$, and

$$
\begin{aligned}
\epsilon_{\nu i} &\sim \text{Normal}(0, \sigma_\nu) && (22)\\
\epsilon_{ri} &\sim \text{Normal}(0, \sigma_r), && (23)
\end{aligned}
$$

for $i = 1 \ldots 243$.

The following priors complete the model specification:

$$\sigma_r \sim \text{Half-normal}(0, 0.2) \tag{24}$$
$$\sigma_d \sim \text{Half-normal}(0, 0.2) \tag{25}$$
$$-\log(\phi_q) \sim \text{Half-normal}(13, 0.5) \tag{26}$$
$$\phi \sim \text{Beta}(7, 77) \tag{27}$$
$$\eta_y \sim \text{Half-normal}(0, 5) \tag{28}$$
$$\eta_q \sim \text{Half-normal}(0, 5) \tag{29}$$
$$T_\rho \sim \text{Gamma}(8.3, 8.3) \tag{30}$$
$$\rho = (0.87T_\rho)^{-1} \tag{31}$$
$$T_\delta \sim \text{Gamma}(10, 10) \tag{32}$$
$$\delta = (97T_\delta)^{-1} \tag{33}$$
$$\gamma' \sim \text{Gamma}(100, 100) \tag{34}$$
$$\gamma = 3.5\gamma' \tag{35}$$
$$d_0' \sim \text{Gamma}(10,\ 10) \tag{36}$$
$$d_0 = 1.47d_0' \tag{37}$$
$$\nu_0 \sim \text{Normal}(0, 0.5) \tag{38}$$
$$r_0 \sim \text{Normal}(0, 0.5) \tag{39}$$
$$\log(V_{N0}) \sim \text{Normal}(0.7, 0.3) \tag{40}$$
$$S_0' \sim \text{Beta}(4, 6) \tag{41}$$
$$E_0' \sim \text{Gamma}(10, 0.1) \tag{42}$$
$$I_0' \sim \text{Gamma}(6, 0.1) \tag{43}$$
$$E_0 = E_0'/N \tag{44}$$
$$I_0 = I_0'/N \tag{45}$$
$$S_0 = S_0'(1 - E_0 - I_0) \tag{46}$$
$$R_0 = 1 - S_0 - E_0 - I_0 \tag{47}$$

Details of prior specification

- **Period of cross-immunity** (T_δ): Reich *et al.* [1] estimate a mean of 1.88 years (97 weeks) with a 95% confidence interval of (0.88, 4.31). We specify a gamma prior on the multiplicative scale to have a mean of one and 0.01 and 0.99 quantiles at roughly $0.88/1.88 = 0.47$ and $4.31/1.88 = 2.3$, respectively:
$$T_\delta \sim \text{Gamma}(10, 10) \tag{48}$$
where $\delta = (97T_\delta)^{-1}$. Note that we use a slightly more conservative interval than Reich *et al.* to avoid numerical instability at extreme values.

- **Intrinsic incubation period** (T_ρ): Chan and Johansson [2] estimate a mean of 6.1 days with a 95% credible interval of (3, 10). Thus, we specify a gamma prior on the multiplicative scale to have a mean of one, and 0.05 and 0.95 quantiles of $3/6.1 = 0.49$ and $10/6.1 = 1.6$, respectively:
$$T_\rho \sim \text{Gamma}(8.3, 8.3) \tag{49}$$

where $\rho = (0.87T_\rho)^{-1}$.

- **Decay rate of infectiousness** (γ): Nguyet *et al.* [3] find that there is very little transmission of dengue from humans to mosquitoes after 5-6 days since the onset of fever (with roughly an extra day of infectiousness prior to onset of fever). In reality, an individual's infectiousness decays with time, but the ODE model assumes infectiousness is constant for the (exponentially distributed) duration of the infection period. To parameterize the model, we assume a rough equivalence between the probability of infection from a single individual at a given time and the proportion of individuals still infectious at a given time. Thus we choose our prior for γ to keep the time at which only 5% of individuals are still infectious between 5 and 7 days, with a mean 5% threshold of 6 days. Again we specify the prior on the multiplicative scale to have a mean of one and 0.05 and 0.95 quantiles at $2.99/3.5 = 0.85$ and $4.19/3.5 = 1.2$, respectively:

$$\gamma' \sim \text{Gamma}(100, 100) \tag{50}$$

where $\gamma = 3.5\gamma'$.

- **Baseline mosquito mortality rate** (d_0): Brady *et al.* [4] estimated *Aedes aegypti* temperature-dependent field survivorship curves from over 190 laboratory and field experiments. They found that increased mortality in the field generally washes out the effect of temperature, so we use their estimates for 25 degrees C to construct our prior for d_0. We use nonlinear least squares to fit the exponential cdf with rate d_0 to the estimated mortality curve provided by Brady *et al.*, which gives $d_0 = 1.47$. We specify a gamma prior on the multiplicative scale, with parameters chosen to keep the prior mass within a factor of 2 of the mean:

$$d_0' \sim \text{Gamma}(10, 10) \tag{51}$$

where $d_0 = 1.47d_0'$.

- **Initial conditions** (S_0, E_0, I_0, R_0): The SEIRS model assumes a constant population size and is scaled so that $S_0 + E_0 + I_0 + R_0 = 1$. We place priors on the initial number of exposed and infectious individuals, rather than the initial proportion, as it provides a more intuitive scale. We set the prior mean for E_0' and I_0' at 100 and 60 individuals respectively, to account for the fact that the annual epidemic should be just beginning, with infectious individuals lagging behind exposed. These means were chosen large enough to ensure sustained multi-annual transmission, but with a relatively large variance. We choose the prior for S_0', the proportion of remaining individuals initially susceptible, to have a mean of roughly 0.4, with the mass concentrated between 0.2 and 0.6. This range produces dynamics with recurring annual outbreaks and avoids the extremes where the disease either does not spread or spreads far too quickly and then dies out. This is also roughly consistent with [5] who report notified cases for Vitoria from 1995 to 2008. They report that the 2008 outbreak (where our data begins) was driven by serotypes 2 and 3, and 2009 by serotype 2 only, with 13590 reported serotype 2 cases in previous years. Assuming 12 unreported cases for every reported case, there should be 163,080 total cases, or about 50% of the population, immune to serotype 2 at the start of 2008.

- **Initial conditions** (ν_0, r_0): Since the harmonic oscillators on ν and r are centered at zero, and we do not have any prior knowledge of where the oscillator should be initially, we center the priors of their initial conditions at zero as well. Further, since r is a population growth rate for a relatively stable (i.e., not explosive) population, it should not be too far from zero. Similarly, ν, as an exponential multiplier on the mean per-capita mortality, should not vary too far from zero either. As such, we use flexible, but still conservative prior standard deviations of 0.5 for both values.

- **Mosquito population size and capture probability** ($\log(V_{N0}), \phi_q$): Wearing and Rohani [6] assume 2 mosquitoes per person in their model of dengue transmission, so we use that to inform our prior on $\log(V_{N0})$. Since V_N has been scaled by the human population size, we center the prior for $\log(V_{N0})$ at $\log(2) = 0.7$ and set the variance to concentrate mass between 0.5 and 5 mosquitoes per person. The per-trap mosquito capture probability, ϕ_q follows V_{N0} closely and scales the estimated mosquito abundance down to magnitude of the trap counts. Since $E[q_t] = \phi_q \tau V_{Nt} N$, we set the prior on $\log(\phi_q)$ to have a mean of $\log(q_1) - \log(\tau_1) - \log(V_{N0}) - \log(N) = -13$ and a variance to match the prior on $\log(V_{N0})$.

- **Case reporting probability** (ϕ): Silva *et al.* [7] estimate a reporting rate of 1/12 (0.083) in Brazil. Because the reporting rate is bound between 0 and 1, we used a Beta-distribution for our prior, parameterized to have a mean of 1/12, with most of the mass between 1/6 and 1/24:
$$\phi \sim \text{Beta}(7, 77) \tag{52}$$

- **Mosquito process variances** (σ_r, σ_d): For the purposes of prediction and generalization, we want latent growth and death time series to be smooth and regularly oscillating. To achieve this, we put a relatively strong, half-normal prior on the process standard deviations. A prior standard deviation of 0.2 provides flexibility while still pulling the ϵ terms toward zero.

- **Overdispersion parameters** (η_y, η_q): The negative binomial likelihoods were parameterized as:
$$\text{NegBin}(y|\mu, \eta) = \binom{y + 1/\eta - 1}{y} \left(\frac{\mu\eta}{1 + \mu\eta}\right)^y \left(\frac{1}{1 + \eta\mu}\right)^{1/\eta} \tag{53}$$
such that
$$E[y] = \mu \tag{54}$$
$$\text{Var}[y] = \mu + \eta\mu^2 \tag{55}$$
Thus, $\eta = 0$ corresponds to Poisson, and $\eta > 0$ leads to overdispersion. We put a relatively conservative truncated normal prior on both η_y and η_q that concentrates mass near zero but allows for larger values.

Fixed parameters

- **Transmission rate** (λ): The transmission rate only appears multiplied by a term from the mosquito model (either V_I or V_S) and is thus effectively non-identifiable when we are also estimating the overall size of the mosquito population. Because of this, we fix λ at a reasonable value from the literature (though our results should not be sensitive to the exact value used). The transmission rate is the product of the mosquito feeding rate and the probability of transmission given feeding. Scott *et al.* [8] estimate that *Aedes aegypti* females take 0.63 blood meals per day in Puerto Rico, and 0.76 blood meals per day in Thailand. We take the average of these and set the biting rate at 0.69 per day, or 4.87 per week. There are relatively few reliable estimates of the transmission probability of DENV, but Lambrechts *et al.* [9] estimate transmission probabilities close to one for several related flaviviruses at 25 degrees C (roughly the mean temperature for Vitoria).

- **Extrinsic incubation period** ($1/\rho_v(t)$): Temperature is thought to play an important role in the seasonality of dengue, in particular through its influence on the extrinsic incubation period of the virus in the mosquito (the amount of time until a mosquito becomes infectious after taking an infectious blood meal). To account for this, we make $1/\rho_v$ a function of temperature, and force it with weekly mean temperature records for Vitoria downloaded from Weather Underground (Goiabeiras weather station, station ID: IESVITOR2). We use the temperature relationship from the meta-analysis of Chan annd Johansson [2], which incorporates a large number of studies and accounts for the interval-censored nature of the data usually available from mosquito transmission studies. Chan and Johansson find that the log-normal and gamma distributions are nearly indistinguishable in providing the best fit to the EIP data. To capture a gamma-distributed EIP in the differential equation model, we follow [10] and chain together ν exposed compartments, each with an out-flow rate of $\nu\rho_v(t)$ to generate

$$EIP \sim \text{Gamma}(\nu, \nu\rho_v(t)) \tag{56}$$

such that $E[EIP] = 1/\rho_v(t)$. From [2], we let $\nu = 4$ and

$$\rho_v(t) = 7\exp(0.21T(t) - 7.9) \tag{57}$$

This relationship is similar to those reported in [11] and [12], though there is uncertainty about the exact shape of this relationship.

References

[1] Reich NG, Shrestha S, King Aa, Rohani P, Lessler J, Kalayanarooj S, et al. Interactions between serotypes of dengue highlight epidemiological impact of cross-immunity. J R Soc Interface. 2013;10(86):20130414. doi:10.1098/rsif.2013.0414.

[2] Chan M, Johansson MA. The incubation periods of dengue viruses. PLoS One. 2012;7(11):1–7. doi:10.1371/journal.pone.0050972.

[3] Nguyet MN, Duong THK, Trung VT, Nguyen THQ, Tran CNB, Long VT, et al. Host and viral features of human dengue cases shape the population of infected and infectious *Aedes aegypti* mosquitoes. Proc Natl Acad Sci U S A. 2013;110(22):9072–7. doi:10.1073/pnas.1303395110.

[4] Brady OJ, Johansson MA, Guerra CA, Bhatt S, Golding N, Pigott DM, et al. Modelling adult *Aedes aegypti* and *Aedes albopictus* survival at different temperatures in laboratory and field settings. Parasit Vectors. 2013;6:351. doi:10.1186/1756-3305-6-351.

[5] Cardoso I, Cabidelle A. Dengue: clinical forms and risk groups in a high incidence city in the southeastern region of Brazil. Revista da Sociedade Brasileira de Medicina Tropical. 2011;44(4):430–435.

[6] Wearing HJ, Rohani P. Ecological and immunological determinants of dengue epidemics. Proc Natl Acad Sci U S A. 2006;103(31):11802–7. doi:10.1073/pnas.0602960103.

[7] Silva MMO, Rodrigues MS, Paploski IAD, Kikuti M, Kasper AM, Cruz JS, et al. Accuracy of dengue reporting by national surveillance system, Brazil. Emerg Infect Dis. 2016;22(2):336–339. doi:10.3201/eid2202.150495.

[8] Scott TW, Amerasinghe PH, Morrison AC, Lorenz LH, Clark GG, Strickman D, et al. Longitudinal studies of *Aedes aegypti* (Diptera: Culicidae) in Thailand and Puerto Rico: blood feeding frequency. J Med Entomol. 2000;37(1):89–101.

[9] Lambrechts L, Paaijmans KP, Fansiri T, Carrington LB, Kramer LD, Thomas MB, et al. Impact of daily temperature fl uctuations on dengue virus transmission by Aedes aegypti. Proc Natl Acad Sci U S A. 2011;108(18):1–6. doi:10.1073/pnas.1101377108/-/DCSupplemental.www.pnas.org/cgi/doi/10.1073/pnas.1101377108.

[10] Lloyd AL. Realistic distributions of infectious periods in epidemic models: Changing patterns of persistence and dynamics. Theor Popul Biol. 2001;60(1):59–71. doi:10.1006/tpbi.2001.1525.

[11] Focks Da, Daniels E, Haile DG, Keesling JE. A simulation model of the epidemiology of urban dengue fever: literature analysis, model development, preliminary validation, and samples of simulation results. Am J Trop Med Hyg. 1995;53(5):489–506.

[12] Tjaden NB, Thomas SM, Fischer D, Beierkuhnlein C. Extrinsic Incubation Period of Dengue: Knowledge, Backlog, and Applications of Temperature Dependence. PLoS Negl Trop Dis. 2013;7(6):1–5. doi:10.1371/journal.pntd.0002207.
